# Factors associated with inadequate follow-up of children with congenital syphilis

**DOI:** 10.11606/s1518-8787.2019053001284

**Published:** 2019-10-16

**Authors:** Ana Nery Melo Cavalcante, Maria Alix Leite Araújo, Marina Arrais Nobre, Rosa Lívia Freitas de Almeida

**Affiliations:** I Universidade de Fortaleza. Faculdade de Medicina. Programa de Pós-Graduação em Saúde Coletiva. Fortaleza, CE, Brasil; II Universidade de Fortaleza. Centro de Ciências da Saúde. Programa de Pós-Graduação em Saúde Coletiva. Fortaleza, CE, Brasil; III Universidade de Fortaleza. Curso graduação em Medicina. Fortaleza, CE, Brasil

**Keywords:** Congenital syphilis, pharmacological treatment, Secondary Health Care, Loss of follow-up, Risk factors

## Abstract

**OBJECTIVE:**

To analyze factors associated with outpatient follow-up of children with congenital syphilis.

**METHODS:**

A non-concurrent cohort study performed in primary care units and three reference maternity hospitals in Fortaleza (Ceará State). Data were collected from September 2013 to September 2016 in the notification forms and in the medical records of hospitalization and outpatient follow-up, and they were presented considering an adequate and inadequate follow-up. Children who attended the primary care unit or referral outpatient clinic during the period recommended by the Ministry of Health were considered adequately followed up and performed the recommended examinations. Pearson’s chi-square and Fisher’s exact tests were used in the comparative analysis. The estimated risk of adequate non-follow-up was verified by simple and multiple logistic regression.

**RESULTS:**

The total of 460 children with congenital syphilis were notified, of which 332 (72.2%) returned for at least one appointment and were part of the study. Exactly 287 (86.4%) children attended the primary health unit; however, there was no reference to congenital syphilis in 236 (71.1%) medical records and no information on the venereal disease research laboratory (VDRL) test was found in 264 (79.5%) children. There was nonadherence to subsequent appointments by 272 (81.9%) individuals. The following variables had a statistically significant association with the non-adequate follow-up of the children: marital status of the mothers, number of prenatal appointments, number of pregnancies, blood count, and radiography of long bones.

**CONCLUSIONS:**

Most children with congenital syphilis attended primary care for follow-up, but the services do not meet the recommendations of the Brazilian Ministry of Health for adequate follow-up.

## INTRODUCTION

Congenital syphilis (CS) is transmitted from the infected pregnant woman to the conceptus by the bacterium *Treponema pallidum* via placenta or through the infant’s contact with active lesions in the birth canal. The highest probability of transmission occurs when pregnant women are in the primary and secondary stages of the infection^1^ . Untreated syphilis in pregnant women can cause (40% of cases) miscarriage, fetal death, neonatal death, and, when children survive, about 20% are symptomatic and present early (under two years) and late (over two years) manifestations^2 , 3^ .

The World Health Organization estimates that approximately 1.3 to 2.0 million pregnant women are infected each year with syphilis^4^ . In Brazil, the mean prevalence of gestational syphilis ranges from 1.4% to 2.8%, resulting in 25% of vertical transmission rate^5^ .

In 2010, the Pan American Health Organization member states, including Brazil, approved the Strategy and Plan of Action for Elimination of Mother-to-child Transmission of HIV and Syphilis. The goal was to reduce the CS incidence to less than or equal to 0.5 cases per 1,000 live births (LB) by 2015^6^ . However, we observed that an increase in the incidence rate between 2010 and 2016, ranging from 2.4 cases per 1,000 LB to 6.8 per 1,000 LB^7^ . Despite the fact that CS has been mandatory since 1986, there is considerable underreporting^5^ .

In Ceará, the number of CS notifications between 2010 and 2016 increased by 67.3%. In 2016, the incidence rate was 10.2 cases per 1,000 LB, the highest in the period. In that same year, in the municipality of Fortaleza, there was a detection rate of 20.9 cases per 1,000 LB^8^ .

CS is considered a sentinel event, once it is preventable as long as health actions are efficient. The Brazilian Ministry of Health (MH) recommends the further investigation of these in order to identify weaknesses in care, as well as strategies to overcome them^9^ . In Brazil, although prenatal coverage is greater than 90%^10^ and the availability of benzathine penicillin – a low cost drug that is easy to apply and has no scientific evidence of resistance cases –, most cases of syphilis in pregnant women are inadequately treated, resulting in prolonged and costly treatment and unfavorable outcomes in children^11^ .

All children exposed to CS during pregnancy, even with properly treated mothers, should receive follow-up with monthly outpatient appointments until the 6th month of life and bimonthly from the 6th to the 18th month. Its control is performed by the venereal disease research laboratory (VDRL) examination in children, at 1, 3, 6, 12 and 18 months of age, and it can be interrupted after two consecutive negative tests. Semi-annual ophthalmic, neurological and audiological assessment is also required for two years. In children whose cerebrospinal fluid (CSF) at birth has shown abnormal results, CSF analysis should be repeated every six months until normalization of biochemical (protein), cytological and immunological parameters (VDRL titration)^12^ .

Adequate follow-up of CS is essential to avoid complications and late sequelae in children. Studies evaluating the follow-up of children exposed or diagnosed with CS ^13 - 15^ were conducted in referral services and found a high loss to follow-up rate.

Given the above, this study aimed to analyze factors associated with the inadequate follow-up of children with CS in primary health care units and specialized outpatient clinics of referral maternities.

## METHODS

A non-concurrent cohort study conducted in Fortaleza, Ceará State, in primary care units and municipal public maternity hospitals that have a referral outpatient clinic in infectology and follow-up of children with CS. During the study period, there were four municipal maternity hospitals with specialized outpatient clinics, but the collection was conducted only in three, since it was not possible to access the medical records in one of these services.

Initially, data from September 2013 to September 2016 were collected in the CS notification forms of each maternity hospital, complemented by information from medical records of hospitalization and children outpatient follow-up. Subsequently, information on the follow-up of children in primary care was collected using the Fastmedic^®^ electronic medical record of the Municipal Health Secretariat.

In 2017, these maternity hospitals performed 25% of deliveries and reported 23.8% of CS cases in the municipality. All of these hospitals provide treatment for children with CS in the neonatal period, as well as specialized outpatient follow-up after hospital discharge.

This study included all children notified with CS in the study period, regardless of the mothers’ treatment. Those who died during hospitalization and those who did not return after hospital discharge were excluded due to the need to assess follow-up variables.

The maternal variables analyzed were: sociodemographic (age, marital status and schooling) and gestational variables (prenatal care, number of appointments, quarter of the beginning of prenatal care, number of pregnancies, time of diagnosis, maternal treatment, partner’s treatment and human immunodeficiency virus (HIV) co-infection. The variables related to children were: weight classification according to gestational age, gestational age at birth, VDRL titration at birth, presence of signs and symptoms, and long-term blood count, CSF, and long-bone radiography (X-ray) results.

Children were considered symptomatic when they had at least one of the following conditions: prematurity, low birth weight, hepatomegaly with or without splenomegaly, skin lesions, respiratory distress with pneumonia, serosanguinolent rhinitis or indirect bilirubin jaundice with indication level for phototherapy or for direct bilirubin. A complete blood count (CBC) was considered abnormal when anemia, thrombocytopenia, leukocytosis, or leukopenia were present. CSF, if it had proteins > 150 mg/dl, cellularity > 25 cells/mm^3^ or reactive VDRL. Alterations in long-bone X-ray were considered osteitis, periostitis or osteochondritis^12^ .

The information collected about the children’s follow-up were: VDRL results at 1, 3, 6, 12 and 18 months of age, number of appointments, clinical changes, sequelae, retreatment and age at which they presented two consecutive negative VDRL results.

Data were presented according to the parent and child variables and arranged in two groups, according to CS follow-up: adequate and inadequate. The children who attended the primary care unit or the referral outpatient clinic during the period recommended by the MH and performed the recommended exams were considered adequately followed^12^ .

Data were analyzed using the Statistical Package for Social Sciences (SPSS) version 23. Pearson’s chi-square and Fisher’s exact tests were used in the comparative analysis. The estimated risk of inadequate follow-up was verified by simple and multiple logistic regression considering the 95% confidence interval. Variables with p-values inferior than 0.2 in the univariate analysis^16^ were considered in the multiple logistic regression. In the model, the variables selection was performed by likelihood-ratio test and the Wald test, at 0.05 significance level. The final model was measured based on the percentage improvement model in relation to the initial deviance (likelihood-ratio). Throughout the analysis, p < 0.05 was considered significant as a condition for rejecting the null hypothesis. The research was approved by the Research Ethics Committee of the Universidade de Fortaleza (Opinion No. 2.505.247) and conducted within ethical standards.

## RESULTS

**Figure 1:**
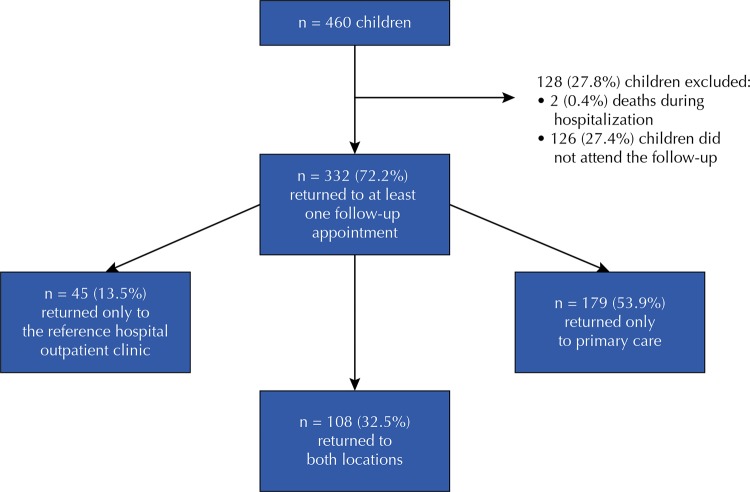
Graphical representation of the cohort with exclusion criteria and follow-up locations of children notified with congenital syphilis who returned to at least one follow-up appointment. Fortaleza, CE, 2013–2016.

During the study period, 460 children with CS were reported in the three maternity wards. In total, 126 (27.4%) children did not attend any follow-up appointment, of which 87 (69%) were not registered in any primary care unit in the city of Fortaleza. Therefore, 332 (72.2%) children who returned for at least one appointment were part of the study, as shown in Figure 1 . In two consecutive VDRL tests, 60 children (18.1%) had negative results and adequate follow-up, which occurred with 3.8 months of mean age (minimum of 3 and maximum of 12 months). There was no adherence to follow-up by 272 (81.9%) children. The mean of appointments in the reference maternity hospital was 3.3 (SD = 2.6; minimum of 1 and maximum of 12) and in primary care, 3.6 (SD = 3.3; minimum of 1 and maximum of 22).

Of the 332 children who returned for at least one follow-up appointment, 287 (86.4%) were seen at the primary care; however, there were no references to CS in 236 (71.1%) medical records and no information about VDRL request was found in 264 (79.5%).

**Figure 2:**
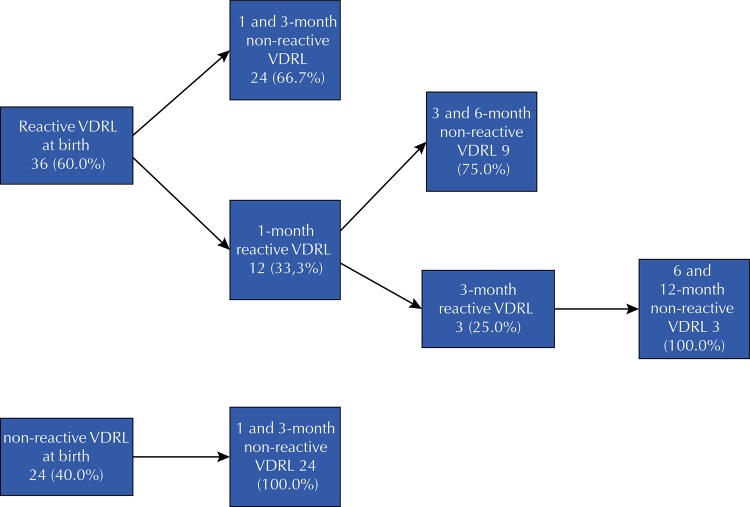
VDRL evolution in children with adequate follow-up for congenital syphilis (n = 60). Fortaleza, CE, 2013–2016.

Among the 60 (18.1%) children who underwent adequate follow-up, 47 (78.3%) attended the referral outpatient clinic or the primary care unit. No child was attended exclusively in primary care. Also, 36 children (60%) showed reactive VDRL at birth, and none were superior to maternal VDRL at two dilutions. Of these children, 33 (91.7%) had negative results in both VDRL until the sixth month. In this group, no child was indicated for retreatment, as shown in Figure 2 . Regarding the group that did not adhere to follow-up, we found six (2.2%) children with neuropsychomotor developmental delay (NPMD), one of whom was followed in the specialized service and five in primary care.

VDRL: Venereal Disease Research Laboratory test

Table 1 presents the mothers’ sociodemographic information regarding the children’s follow-up. The mothers’ mean age was 23.9 years (SD = 6.3; minimum of 14 and maximum of 43 years). Children whose mothers were single were 1.9 times more likely (p = 0.037; 95%CI 1.0–3,5) to lack the adequate follow-up when compared with those whose mothers were married or living in a stable union, an effect that disappeared in the adjusted analysis.

**Table 1:** Univariate logistic regression of maternal sociodemographic characteristics associated with inadequate follow-up of children reported with congenital syphilis. Fortaleza, CE, 2013–2016.

Characteristics	n (%)	Adequate follow-up		p	Crude OR (95%CI)

		No	Yes		
	
		n (%)	n (%)		
Age (years)				0.958	
< 20	95 (28.6)	78 (82.1)	17 (17.9)		1
≥ 20	237 (71.4)	194 (81.9)	43 (18.1)		1.0 (0.5–1.8)
Marital status^b^				0.037	
Married/stable union	188 (57.3)^a^	147 (78.2)	41 (21.8)		1
Single	140 (42.7)^a^	122 (87.1)	18 (12.9)		1.9 (1.0–3.5)
Years of Schooling				0.853	
< 8	225 (68.8)^a^	185 (82.2)	40 (17.8)		1.1 (0.6–1.9)
≥ 8	102 (31.2)^a^	83 (81.4)	19 (18.6)		1

^a^ Values differ in each variable because ignored data have not been considered for analysis.

^b^ In multivariate analysis, this variable did not remain significant and the adjusted OR was not added.

Table 2 shows maternal information on pregnancy, prenatal care, treatment of syphilis, and HIV co-infection. A total of 297 pregnant women attended prenatal care (89.5%), with a mean of 5.4 appointments. The odds of not returning for follow-up were 1.8 times higher (p = 0.042; 95%CI 1.1–3.2) among children whose mothers had less than six prenatal appointments and 2.0 times higher (p = 0.018; 95%CI 1.1–3.5) among children whose mothers had more than one pregnancy.

**Table 2:** Simple and multiple logistic regression of prenatal characteristics, diagnosis of gestational syphilis, treatment of the pregnant woman and her partner, and HIV co-infection associated with inadequate follow-up of children reported with congenital syphilis. Fortaleza, CE, 2013–2016.

Characteristics	n (%)	Adequate follow-up		p	Crude OR (95%CI)	Adjusted OR^b^ (95%CI)

		No	Yes			
	
		n (%)	n (%)			
Prenatal care				0.880		
No	35 (10.5)	29 (82.9)	6 (17.1)		1.1 (0.4–2.7)	
Yes	297 (89.5)	243 (81.8)	54 (18.2)		1	
Number of medical appointments				0.042		
< 6	155 (46.8)^a^	134 (86.5)	21 (13.5)		1.8 (1.1–3.2)	
≥ 6	176 (53.2)^a^	137 (77.8)	39 (22.2)		1	
Quarter of the beginning of Prenatal care				0.859		
1^st^	131 (44.7)^a^	107 (81.7)	24 (18.3)		1	
2^nd^/3^rd^	162 (55.3)^a^	131 (80.9)	31 (19.1)		0.9 (0.5–1.7)	
Number of pregnancies				0.018		
1	96 (29.1)^a^	71 (74.0)	25 (26.0)		1	1
> 1	234 (70.9)^a^	199 (85.0)	35 (15.0)		2.0 (1.1–3.5)	2.1 (1.1–3.8)
Moment of diagnosis				0.566		
Prenatal care	239 (72.0)	194 (81.2)	45 (18.8)		1	
Delivery/Postpartum	93 (28.0)	78 (83.9)	15 (16.1)		1.2 (0.6–2.3)	
Prenatal treatment				0.707		
Adequate	14 (4.2)	12 (85.7)	2 (14.3)		1	
Inadequate/Not treated	318 (95.8)	260 (81.8)	58 (18.2)		0.7 (0.2–3.4)	
Treated Partner				0.780		
No	278 (84.3)^a^	227 (81.7)	51 (18.3)		0.9 (0.4–2.0)	
Yes	48 (14.7)^a^	40 (83.3)	8 (16.7)		1	
HIV co-infection				0.408		
No	322 (99.1)^a^	262 (81.4)	60 (18.6)		–	
Yes	3 (0.9)^a^	3 (100)	0 (0)		–	

^a^ Values differ in each variable because ignored data have not been considered for analysis.

^b^ Variables that remained significant after multivariate analysis.

Table 3 shows the children’s variables. Those with abnormal blood count and no long-bone X-ray were 1.8 times (p = 0.049; 95% CI 1.0–3.5) and 5.7 times (p = 0.006; 95%CI 1.7–18.8) more likely to not return for follow-up, respectively, than children with normal blood count and X-ray.

**Table 3:** Simple and multivariate logistic regression of the variables weight classification for gestational age, gestational age at birth, VDRL at birth, clinical symptoms, blood count, CSF, and long-bone X-ray associated with inadequate follow-up of children reported with congenital syphilis. Fortaleza, CE, 2013–2016.

Characteristics	n (%)	Adequate follow-up		p	Crude OR (95%CI)	Adjusted OR^b^ (95%CI)

		No	Yes			
	
		n (%)	n (%)			
Weight classification for gestational age				0.343		
AGA	259 (78.5)^a^	211 (81.5)	48 (18.5)		1	
SGA	43 (13.0)^a^	38 (88.4)	05 (11.6)		1.7 (0.6–4.6)	
LGA	28 (8.5)^a^	21 (75.0)	07 (25.0)		0.6 (0.3–1.6)	
Gestational age at birth						
Preterm	30 (9.1)^a^	24 (80.0)	06 (20.0)		1	
Term	300 (90.9)^a^	246 (82.0)	54 (18.0)		1.2 (0.4–2.9)	
VDRL at birth				0.138		
Non-reactive	106 (31.9)	82 (77.4)	24 (22.6)		1	
Reactive	226 (68.1)	190 (84.1)	36 (15.9)		1.5 (0.9–2.7)	
Presence of clinical signs and symptoms				0.538		
No	244 (73.5)	198 (81.1)	46 (18.9)		0.8(0.4–1.5)	
Yes	88 (26.5)	74 (84.1)	14 (15.9)		1	
Complete blood count				0.049		
Normal	173 (59.9)^a^	133 (76.9)	40 (23.1)		1	1
Altered	116 (40.1)^a^	100 (86.2)	16 (13.8)		1.8 (1.0–3.5)	2.0(1.1–3.9)
CSF cytology and/or proteins						
Normal	189 (57.6)^a^	152 (80.4)	37 (19.6)	0.224	1	
Altered	30 (9.1)^a^	28 (93.3)	02 (6.7)		3.4 (0.8–14.6)	
Unexecuted	109 (33.3)^a^	88 (80.7)	21 (19.3)		1.0 (0.5–1.6)	
VDRL in CSF						
Non-active	219 (66.2)^a^	180 (82.2)	39 (17.8)	0.833	1	
Unexecuted	112 (33.8)^a^	91 (81.2)	21 (18.8)		0.9 (0.5–1.7)	
Long-bone x-ray						
Normal	256 (79.0)^a^	200 (78.1)	56 (21.9)	0.006	1	1
Altered	4 (1.2)^a^	03 (75.0)	01 (25.0)		0.7 (0.1–6.6)	
Unexecuted	64 (19.8)^a^	61 (95.3)	03 (4.7)		5.7 (1.7–18.8)	4.9(1.5–16.4)

AGA: adequate for gestational age; SGA: small for gestational age; LGA: large for gestational age; CSF: cerebrospinal fluid; X-ray: radiography; VDRL: venereal disease research laboratory test

^a^ Values differ in each variable because ignored data have not been considered for analysis.

^b^ Variables that remained significant after multivariate analysis.

In total, 332 children performed the VDRL at birth (100%), 289 performed the CBC (87.0%), 219 the CSF cytology/protein (66.7%), 219 the VDRL of CSF (66.2%), and 260 the long-bone X-ray (80.2%). The mean cohort weight was 3,160 g, ranging from 940 g to 5,160 g. The total of 37 (11.2%) children were born with low birth weight (< 2,500 g). Regarding gestational age at birth, 30 (9.1%) were born preterm (< 37 weeks), with no significant association with the follow-up outcome.

In multivariate logistic regression analysis, having more than one pregnancy, no long-bone X-rays and altered blood count remained implicated in the inadequate follow-up outcome of children with congenital syphilis.

## DISCUSSION

In this study, we can observe serious problems regarding the outpatient follow-up of children with CS. Considerable proportion of children attended the primary care unit, but most did not have CS investigated at this level of care. This fact shows the poor visibility and recognition of CS as an important public health problem, either due to the lack of professionals’ awareness or because they feel that the follow-up of these children is not a primary care issue.

In Fortaleza, primary care works through the Family Health Strategy (FHS), and most pregnant women with syphilis attend prenatal care in these units, where they most likely take the infant for follow-up. It is noteworthy that the professionals did not consider the mother’s previous history, her syphilis diagnosis in pregnancy and the child’s birth history, missing opportunities to assess and prevent severe forms of CS.

Even with children’s attendance at some health service, the proportion of nonadherence to follow-up was high, a situation also evidenced in other studies on this subject^13^ . Surveys that assessed the follow-up of children with CS were conducted in referral outpatient clinics and did not consider the reasons for poor adherence. We believe the distance from the unit to the residence contributes to the absence in these services.

We emphasize that children also did not return to primary health care units. Possibly, mothers initially sought these units to perform childcare, and professionals missed the opportunity to properly refer the cases. The low adherence to childcare may have occurred due to the children having no health problems that justify, for the mother, the need to attend the unit frequently. There seems to be a misconception that children with CS, as they have no symptoms, do not need follow-up, a situation corroborated by a study that assessed childcare in the South and Northeast regions of Brazil, which found that adequate follow-up of children occurred only in 20% of cases^17^ .

The poor adherence to follow-up could be minimized if health professionals, both at the hospital discharge time and at the child’s first appointment, were careful to advise mothers about the importance of follow-up, reinforcing attendance at subsequent appointments, especially in the CS case, in which most children are born without symptoms.

These findings indicate the need to reflect on the health care network organization, especially regarding the definition of appropriate places to accompany children with CS after hospital discharge. Therefore, it is necessary to define the role of primary care in this process, as well as sensitize and train professionals at this level of care.

At the time of this investigation, all children with CS, when discharged, were guided to the referral outpatient clinics of maternities. Since the conduction of this study, the situation has been changing, and the municipality is currently discussing the organization of the healthcare network with the definition of appropriate places for the follow-up of these children and ensuring care, active search and referral and counter-referral.

The city of Fortaleza adopts as a public policy the link of all children to a primary care unit. They should receive home visits from FHS professionals in order to guide mothers on the importance of attending appointments and assess adherence to recommendations, among other objectives^18^ . It is noteworthy that, even followed up in reference units, children with CS should be monitored by primary care professionals.

In this study, we observed that in both, outpatient maternity and primary healthcare clinics, the mean of appointments was below the recommended by the MH for CS, which is nine appointments in the first year of life^12^ . In Guarapuava, Paraná, 40 follow-ups of children exposed or diagnosed with CS, had a mean of 6.1 appointments in one year^19^ .

Children’s mean age when they presented two consecutive negative VDRL results was 3.8 months. This result was expected since, in properly treated children, VDRL tends to be negative between three and six months of age^12^ . In Porto Alegre, Rio Grande do Sul, a study assessed 119 follow-ups of children with CS and found that, in 81.5%, the VDRL became negative at three months of age^13^ .

It is important to identify maternal sociodemographic aspects that may prevent children attending the service. Children of single mothers returned less for follow-up, which can be attributed to the fact they are providers and find it difficult to leave work, or for other reasons that prevent them from taking their children to appointments, such as lack of financial resources.

We observed significant association between the number of prenatal appointments and poor adherence to follow-up. In the case of mothers who do not adhere to prenatal care, they are also expected to not attend the follow-up of the child. Therefore, considering that the primary care units in Fortaleza act within the logic of the FHS, professionals should have given greater attention to the active search of these women and their children.

A worrying situation was that all children concomitantly exposed to syphilis and HIV did return for follow-up, which should happen in the specialized outpatient clinic, with primary care monitoring. Children exposed to HIV tend to receive more attention from services, which was evidenced in a survey conducted in São Paulo that found greater adherence to follow-up of these children than those exposed and/or diagnosed with CS^14^ .

Women with more than one pregnancy had a higher chance of not taking their children to appointments, a situation similar to that found in Curitiba, Paraná^15^ . Mothers of more than one child find it difficult to go to health care, either because of household chores or the need to find someone who can be responsible for the other children during her absence.

Changes in blood count and failure to perform long-bone X-ray showed a statistically significant association with the non-follow-up of children, a situation that shows serious failures in their effective attachment to services. These cases can effectively present a CS diagnosis and, therefore, deserve greater attention and guidance, guarantees scheduled appointment and active search in case of non-attendance.

Late congenital syphilis is due to early untreated or unhealed CS in children over two years of age^12^ . In this study, children with delayed NPMD in the nonadherent group may have late syphilis sequelae.

Some limitations of this study include the fact that secondary data were analyzed due to the lack of records and incomplete information of some variables. In addition, we did not exclude other clinical conditions that present signs and symptoms similar to CS, reinforcing the importance of further studies on the evolution of clinical findings in children with CS.

## CONCLUSION

Inadequate follow-up of congenital syphilis is related not only to the attitude of mothers to take their children to medical care, but also to the difficulty in performing tests. Most children with CS come to primary care unit, but at this level of care the recommendations of MH for adequate follow-up are not adopted. Therefore, it is necessary to improve the referral and counter-referral system between the different levels of health care, as well as the awareness and training of health professionals, both for an adequate care and more forceful advice on the mother’s responsibility for the infant’s health.
