# Supplementary material for: Factors associated with inadequate follow-up of children with congenital syphilis
Source: Rev Saude Publica. 2019 Oct 16;53:95. doi: 10.11606/s1518-8787.2019053001284 (PMC6802946; doi:10.11606/s1518-8787.2019053001284)
Supplement: Translation

# Translation: Fatores associados ao seguimento não adequado de crianças com sífilis congênita

**Authors:** Ana Nery Melo Cavalcante, Maria Alix Leite Araújo, Marina Arrais Nobre, Rosa Lívia Freitas de Almeida

**Affiliations:** I Universidade de Fortaleza. Faculdade de Medicina. Programa de Pós-Graduação em Saúde Coletiva. Fortaleza, CE, Brasil; II Universidade de Fortaleza. Centro de Ciências da Saúde. Programa de Pós-Graduação em Saúde Coletiva. Fortaleza, CE, Brasil; III Universidade de Fortaleza. Curso graduação em Medicina. Fortaleza, CE, Brasil

**Keywords:** Sífilis Congênita, tratamento farmacológico, Atenção Secundária à Saúde, Perda de Seguimento, Fatores de Risco

## Abstract

**OBJETIVO:**

Analisar fatores associados ao seguimento ambulatorial não adequado de crianças notificadas com sífilis congênita.

**MÉTODOS:**

Estudo de coorte não concorrente, realizado em unidades de atenção primária e três maternidades de referência de Fortaleza (Ceará). Os dados foram coletados de setembro de 2013 a setembro de 2016 nas fichas de notificação e nos prontuários médicos de internamento e de seguimento ambulatorial, e apresentados considerando o seguimento adequado e não adequado. Foram consideradas adequadamente seguidas as crianças que compareceram à unidade de atenção primária ou ao ambulatório de referência no período recomendado pelo Ministério da Saúde e realizaram os exames preconizados. Utilizou-se os testes qui-quadrado de Pearson e exato de Fisher na análise comparativa. O risco estimado de não seguimento adequado foi verificado por regressão logística simples e múltipla.

**RESULTADOS:**

Foram notificadas 460 crianças com sífilis congênita, das quais 332 (72,2%) retornaram para pelo menos uma consulta e fizeram parte do estudo. Compareceram à unidade primária de saúde 287 (86,4%) crianças; entretanto, não havia referência à sífilis congênita em 236 (71,1%) prontuários e não foram encontradas informações acerca da solicitação do exame *venereal disease research laboratory* (VDRL) em 264 (79,5%). Houve não adesão às consultas subsequentes por parte de 272 (81,9%) indivíduos. As seguintes variáveis apresentaram associação estatisticamente significativa com o seguimento não adequado das crianças: estado civil das genitoras, número de consultas no pré-natal, número de gestações, hemograma e radiografia de ossos longos.

**CONCLUSÕES:**

A maioria das crianças notificadas com sífilis congênita comparecem à atenção primária para seguimento, porém os serviços não atendem às recomendações do Ministério da Saúde para o seguimento adequado.

## INTRODUÇÃO

A sífilis congênita (SC) é transmitida da gestante infectada ao concepto pela bactéria *Treponema pallidum* por via transplacentária ou pelo contato do bebê com lesões ativas no canal de parto. A maior probabilidade de transmissão ocorre quando as gestantes se encontram nas fases primária e secundária da infecção^1^ . A sífilis em gestantes, quando não tratada, pode resultar (40% dos casos) em aborto, óbito fetal, óbito neonatal e, quando as crianças sobrevivem, cerca de 20% são sintomáticas e apresentam manifestações precoces (com menos de dois anos de idade) e tardias (acima de dois anos)^2 , 3^ .

A Organização Mundial de Saúde estima que aproximadamente 1,3 a 2,0 milhões de mulheres grávidas são infectadas a cada ano pela sífilis^4^ . No Brasil, a prevalência média da sífilis gestacional varia entre 1,4% e 2,8%, resultando em 25% na taxa de transmissão vertical^5^ .

Em 2010, foi aprovado pelos Estados membros da Organização Pan-Americana da Saúde, dentre eles o Brasil, o Plano de Ação para a Eliminação da Transmissão Vertical do HIV e da Sífilis. A meta era reduzir a incidência de SC para um valor inferior ou igual a 0,5 casos para cada 1.000 nascidos vivos (NV) até o ano de 2015^6^ . Ocorre que, entre os anos de 2010 e 2016, foi observado aumento na taxa de incidência, passando de 2,4 casos por 1.000 NV para 6,8 por 1.000 NV^7^ . Na realidade, esses valores são ainda maiores, uma vez que, apesar de a SC ser de notificação obrigatória desde 1986, existe uma subnotificação considerável^5^ .

No Ceará ocorreu um aumento de 67,3% no número de notificações de SC entre os anos de 2010 e 2016. No ano de 2016, a taxa de incidência foi de 10,2 casos por 1.000 NV, a maior do período. Nesse mesmo ano, no município de Fortaleza, observou-se a taxa de detecção de 20,9 casos por 1.000 NV^8^ .

A SC é considerada um evento sentinela, pois é passível de prevenção desde que as ações de saúde sejam eficientes. A recomendação do Ministério da Saúde (MS) é que os casos sejam investigados com o intuito de identificar as fragilidades no atendimento, bem como estratégias de superação^9^ . No Brasil, apesar de a cobertura do pré-natal ser superior a 90%^10^ e da disponibilidade da penicilina benzatina – droga de baixo custo, fácil aplicação e sem evidências científicas de casos de resistência –, a maioria dos casos de sífilis em gestantes é inadequadamente tratada, tendo como consequência o tratamento prolongado e oneroso e desfechos desfavoráveis nas crianças^11^ .

Todas as crianças expostas à SC na gestação, mesmo com mães adequadamente tratadas, devem receber acompanhamento com consultas ambulatoriais mensais até o 6º mês de vida e bimestrais do 6º ao 18º mês. O seu controle é realizado pelo exame de *venereal disease research laboratory* (VDRL) na criança, com 1, 3, 6, 12 e 18 meses de vida, podendo ser interrompido após dois exames consecutivos negativos. É também necessária a avaliação oftalmológica, neurológica e audiológica semestral por dois anos. Nas crianças em que o líquido cefalorraquidiano (LCR) ao nascimento tenha apresentado resultado alterado, a análise do LCR deve ser repetida a cada seis meses, até a normalização dos parâmetros bioquímicos (proteína), citológicos e imunológicos (titulação do VDRL)^12^ .

O seguimento adequado da SC é fundamental para evitar complicações e sequelas tardias na criança. Estudos que avaliaram o seguimento das crianças expostas ou diagnosticadas com SC^13^ foram realizados em serviços de referência e encontraram elevada taxa de abandono.

Diante do exposto, o objetivo deste estudo foi analisar fatores associados ao seguimento não adequado de crianças notificadas com SC em unidades de atenção primária à saúde e ambulatórios especializados de maternidades de referência.

## MÉTODOS

Estudo de coorte não concorrente, realizado em Fortaleza, Ceará, nas unidades de atenção primária e maternidades públicas municipais que possuem ambulatório de referência em infectologia e realizam o seguimento de crianças com SC. À época deste estudo, existiam quatro maternidades municipais com ambulatório especializado, porém só foi realizada a coleta em três, pois em um desses serviços não foi possível o acesso aos prontuários.

Inicialmente, dados referentes ao período de setembro de 2013 a setembro de 2016 foram coletados nas fichas de notificação de SC de cada maternidade, complementados com informações dos prontuários médicos de internamento e seguimento ambulatorial das crianças. Posteriormente, foram coletadas informações sobre o seguimento das crianças na atenção primária por meio do prontuário eletrônico Fastmedic^®^, da Secretaria Municipal de Saúde.

Essas maternidades realizaram, no ano de 2017, cerca de 25% dos partos e notificaram 23,8% dos casos de SC do município. Todas realizam o tratamento das crianças com SC no período neonatal, bem como o seguimento ambulatorial especializado após a alta hospitalar.

Foram incluídas neste estudo todas as crianças notificadas com SC no período analisado, independentemente do tratamento das mães. Excluíram-se aquelas que evoluíram para óbito durante o internamento e as que não retornaram ao acompanhamento após a alta hospitalar, devido à necessidade de avaliar as variáveis do seguimento.

As variáveis maternas analisadas foram: sociodemográficas (idade, estado civil e escolaridade) e gestacionais (realização do pré-natal, número de consultas, trimestre do início do pré-natal, número de gestações, momento do diagnóstico, realização do tratamento materno, tratamento do parceiro e coinfecção pelo vírus da imunodeficiência humana (HIV). As variáveis relacionadas às crianças foram: classificação do peso conforme a idade gestacional, idade gestacional ao nascimento, titulação do VDRL ao nascer, presença de sinais e sintomas e resultado do hemograma, do LCR e da radiografia (RX) de ossos longos.

As crianças foram consideradas sintomáticas quando apresentavam pelo menos uma das seguintes condições: prematuridade, baixo peso ao nascer, hepatomegalia com ou sem esplenomegalia, lesões cutâneas, sofrimento respiratório com pneumonia, rinite serossanguinolenta ou icterícia por bilirrubina indireta com nível de indicação para fototerapia ou por bilirrubina direta. O hemograma foi considerado alterado quando apresentou anemia, trombocitopenia, leucocitose ou leucopenia. O LCR, se apresentasse proteínas > 150 mg/dl, celularidade > 25 células/mm^3^ ou VDRL reagente. Foram consideradas como alterações na RX de ossos longos a osteíte, periostite ou osteocondrite^12^ .

As informações coletadas sobre o seguimento da criança foram: resultado do VDRL com 1, 3, 6, 12 e 18 meses de vida, número de consultas, alterações clínicas, sequelas, retratamento e idade com a qual apresentou dois VDRL negativos consecutivos.

Os dados foram apresentados segundo as variáveis das genitoras e das crianças e dispostos em dois grupos, conforme o seguimento da SC: adequado e não adequado. Foram consideradas adequadamente seguidas as crianças que compareceram à unidade de atenção primária ou ao ambulatório de referência no período recomendado pelo MS e realizaram os exames preconizados^12^ .

Os dados foram analisados no programa *Statistical Package for Social Sciences* (SPSS) versão 23. Utilizaram-se os testes qui-quadrado de Pearson e exato de Fisher na análise comparativa. O risco estimado de seguimento não adequado foi verificado por regressão logística simples e múltipla considerando o intervalo de confiança de 95%. As variáveis que apresentaram p-valor inferior a 0,2 na análise univariada^16^ foram consideradas na regressão logística múltipla. No modelo, a seleção das variáveis foi realizada pelo teste da razão de verossimilhança e teste de Wald, em nível de significância de 0,05. O modelo final foi aferido com base no percentual de melhoria do modelo com relação à *deviance* inicial (razão de verossimilhança). Em toda a análise foi considerado significante o p < 0,05, como condição para rejeição da hipótese nula. A pesquisa foi aprovada pelo Comitê de Ética e Pesquisa da Universidade de Fortaleza (parecer nº 2.505.247) e conduzida dentro dos padrões éticos.

## RESULTADOS

Durante o período analisado, foram notificadas 460 crianças com SC nas três maternidades. Não compareceram a nenhuma consulta de seguimento 126 (27,4%), das quais 87 (69%) não estavam cadastradas em nenhuma unidade de atenção primária do município de Fortaleza. Fizeram parte do estudo, portanto, 332 (72,2%) crianças que retornaram para pelo menos uma consulta ( Figura 1 ). Sessenta crianças (18,1%) tiveram seguimento adequado e resultado negativo em dois VDRL consecutivos, o que ocorreu com idade média de 3,8 meses (mínimo 3 e máximo 12 meses). Houve não adesão ao seguimento por parte de 272 (81,9%) crianças. A média de consultas no ambulatório da maternidade de referência foi de 3,3 (DP = 2,6; mínimo de 1 e máximo de 12) e na atenção primária, de 3,6 (DP = 3,3; mínimo de 1 e máximo de 22).

**Figura 1:** Representação gráfica da coorte com critérios de exclusão e locais de seguimento das crianças notificadas com sífilis congênita que retornaram para pelo menos uma consulta de seguimento. Fortaleza, CE, 2013–2016.

Das 332 crianças que retornaram para pelo menos uma consulta de seguimento, havia registro de atendimento na atenção primária para 287 (86,4%); entretanto, não havia nenhuma referência à SC em 236 (71,1%) prontuários e não foram encontradas informações acerca da solicitação do VDRL em 264 (79,5%).

Dentre as 60 (18,1%) crianças que realizaram seguimento adequado, 47 (78,3%) frequentaram o ambulatório de referência das maternidades ou a unidade de atenção primária. Nenhuma foi atendida exclusivamente na atenção primária. Apresentaram VDRL reagente ao nascer 36 crianças (60%), e nenhum foi superior ao VDRL materno em duas diluições. Dessas crianças, 33 (91,7%) negativaram os dois VDRL até o sexto mês. Não foi indicado retratamento a nenhuma criança desse grupo ( Figura 2 ). Em relação ao grupo que não aderiu ao seguimento, foram encontrados registros de seis (2,2%) crianças com atraso no desenvolvimento neuropsicomotor (DNPM), das quais uma foi acompanhada no serviço especializado e cinco na atenção primária.

**Figura 2:** Evolução do VDRL nas crianças com seguimento adequado para sífilis congênita (n = 60). Fortaleza, CE, 2013–2016.

VDRL: *venereal disease research laboratory test*

Na Tabela 1 , estão apresentadas informações sociodemográficas das genitoras em relação ao seguimento das crianças. A idade média das mães foi de 23,9 anos (DP = 6,3; mínimo de 14 e máximo de 43) Crianças cujas mães eram solteiras apresentaram 1,9 vezes mais chances (p = 0,037; IC95% 1,0–3,5) de não realizar seguimento adequado quando comparadas com aquelas cujas mães eram casadas ou viviam em união estável, desaparecendo esse efeito na análise ajustada.

**Tabela 1:** Regressão logística univariada das características sociodemográficas maternas associadas ao não seguimento adequado das crianças notificadas com sífilis congênita. Fortaleza, CE, 2013–2016.

Variável	n (%)	Seguimento adequado		p	OR bruto (IC95%)

		Não	Sim		
	
		n (%)	n (%)		
Faixa etária (anos)				0,958	
< 20	95 (28,6)	78 (82,1)	17 (17,9)		1
≥ 20	237 (71,4)	194 (81,9)	43 (18,1)		1,0 (0,5–1,8)
Estado civil^b^				0,037	
Casada/união estável	188 (57,3)^a^	147 (78,2)	41 (21,8)		1
Solteira	140 (42,7)^a^	122 (87,1)	18 (12,9)		1,9 (1,0–3,5)
Anos de estudo				0,853	
< 8	225 (68,8)^a^	185 (82,2)	40 (17,8)		1,1 (0,6–1,9)
≥ 8	102 (31,2)^a^	83 (81,4)	19 (18,6)		1

^a^Os valores se diferenciam em cada variável devido aos dados ignorados não terem sido considerados para a análise.

^b^Na análise multivariada, esta variável não continuou significativa, não sendo acrescentado o OR ajustado.

As informações maternas sobre gestação, assistência pré-natal, tratamento da sífilis e coinfecção pelo HIV estão dispostas na Tabela 2 . Realizaram o pré-natal 297 gestantes (89,5%), com média de 5,4 consultas. As chances de não retornar para seguimento foram 1,8 vezes maiores (p = 0,042; IC95% 1,1–3,2) entre as crianças cujas mães tiveram menos de seis consultas no pré-natal e 2,0 vezes maiores (p = 0,018; IC95% 1,1–3,5) nos filhos de mães com mais de uma gestação.

**Tabela 2:** Regressão logística simples e múltipla das características relacionadas ao pré-natal, diagnóstico da sífilis gestacional, tratamento da gestante e do parceiro e coinfecção pelo HIV associadas ao não seguimento adequado das crianças notificadas com sífilis congênita. Fortaleza, CE, 2013–2016.

Variável	n (%)	Seguimento adequado		p	OR bruto (IC95%)	OR ajustado^b^(IC95%)

		Não	Sim			
	
		n (%)	n (%)			
Pré-natal				0,880		
Não	35 (10,5)	29 (82,9)	6 (17,1)		1,1 (0,4–2,7)	
Sim	297 (89,5)	243 (81,8)	54 (18,2)		1	
Número de consultas				0,042		
< 6	155 (46,8)^a^	134 (86,5)	21 (13,5)		1,8 (1,1–3,2)	
≥ 6	176 (53,2)^a^	137 (77,8)	39 (22,2)		1	
Trimestre de início do pré-natal				0,859		
1°	131 (44,7)^a^	107 (81,7)	24 (18,3)		1	
2°/3°	162 (55,3)^a^	131 (80,9)	31 (19,1)		0,9 (0,5–1,7)	
Número de gestações				0,018		
1	96 (29,1)^a^	71 (74,0)	25 (26,0)		1	1
> 1	234 (70,9)^a^	199 (85,0)	35 (15,0)		2,0 (1,1–3,5)	2,1 (1,1–3,8)
Momento do diagnóstico				0,566		
Pré-natal	239 (72,0)	194 (81,2)	45 (18,8)		1	
Parto/pós-parto	93 (28,0)	78 (83,9)	15 (16,1)		1,2 (0,6–2,3)	
Tratamento no pré-natal				0,707		
Adequado	14 (4,2)	12 (85,7)	2 (14,3)		1	
Inadequado/não tratou	318 (95,8)	260 (81,8)	58 (18,2)		0,7 (0,2–3,4)	
Parceiro tratado				0,780		
Não	278 (84,3)^a^	227 (81,7)	51 (18,3)		0,9 (0,4–2,0)	
Sim	48 (14,7)^a^	40 (83,3)	8 (16,7)		1	
Coinfecção pelo HIV				0,408		
Não	322 (99,1)^a^	262 (81,4)	60 (18,6)		–	
Sim	3 (0,9)^a^	3 (100)	0 (0)		–	

^a^Os valores se diferenciam em cada variável devido aos dados ignorados não terem sido considerados para a análise.

^b^Variáveis que continuaram significativas após análise multivariada.

Na Tabela 3 , estão apresentadas as variáveis das crianças. Aquelas com alteração no hemograma e que não realizaram RX de ossos longos apresentaram 1,8 vezes (p = 0,049; IC95% 1,0–3,5) e 5,7 vezes (p = 0,006; IC95% 1,7–18,8) mais chances de não retornarem para o seguimento, respectivamente, do que as crianças que apresentaram hemograma e RX normais.

**Tabela 3:** Regressão logística simples e multivariada das variáveis classificação do peso para a idade gestacional, idade gestacional ao nascimento, VDRL ao nascer, sintomas clínicos, hemograma, LCR e RX de ossos longos associadas ao não seguimento adequado das crianças notificadas com sífilis congênita. Fortaleza, CE, 2013–2016.

Variável	n (%)	Seguimento adequado		p	OR bruto (IC95%)	OR ajustado^b^(IC95%)

		Não	Sim			
	
		n (%)	n (%)			
Classificação do peso para a idade gestacional				0,343		
AIG	259 (78,5)^a^	211 (81,5)	48 (18,5)		1	
PIG	43 (13,0)^a^	38 (88,4)	05 (11,6)		1,7 (0,6–4,6)	
GIG	28 (8,5)^a^	21 (75,0)	07 (25,0)		0,6 (0,3–1,6)	
Idade gestacional ao nascimento				0,787		
Pré-termo	30 (9,1)^a^	24 (80,0)	06 (20,0)		1	
Termo	300 (90,9)^a^	246 (82,0)	54 (18,0)		1,2 (0,4–2,9)	
VDRL ao nascer				0,138		
Não reagente	106 (31,9)	82 (77,4)	24 (22,6)		1	
Reagente	226 (68,1)	190 (84,1)	36 (15,9)		1,5 (0,9–2,7)	
Presença de sinais e sintomas clínicos				0,538		
Não	244 (73,5)	198 (81,1)	46 (18,9)		0,8(0,4–1,5)	
Sim	88 (26,5)	74 (84,1)	14 (15,9)		1	
Hemograma				0,049		
Normal	173 (59,9)^a^	133 (76,9)	40 (23,1)		1	1
Alterado	116 (40,1)^a^	100 (86,2)	16 (13,8)		1,8 (1,0–3,5)	2,0(1,1–3,9)
Citologia e/ou proteínas no LCR						
Normal	189 (57,6)^a^	152 (80,4)	37 (19,6)	0,224	1	
Alterado	30 (9,1)^a^	28 (93,3)	02 (6,7)		3,4 (0,8–14,6)	
Não realizado	109 (33,3)^a^	88 (80,7)	21 (19,3)		1,0 (0,5–1,6)	
VDRL no LCR						
Não reagente	219 (66,2)^a^	180 (82,2)	39 (17,8)	0,833	1	
Não realizado	112 (33,8)^a^	91 (81,2)	21 (18,8)		0,9 (0,5–1,7)	
RX dos ossos longos						
Normal	256 (79,0)^a^	200 (78,1)	56 (21,9)	0,006	1	1
Alterada	4 (1,2)^a^	03 (75,0)	01 (25,0)		0,7 (0,1–6,6)	
Não realizada	64 (19,8)^a^	61 (95,3)	03 (4,7)		5,7 (1,7–18,8)	4,9(1,5–16,4)

AIG: adequado para a idade gestacional; PIG: pequeno para a idade gestacional; GIG: grande para a idade gestacional; LCR: líquido cefalorraquidiano; RX: radiografia; VDRL: *venereal disease research laboratory test*

^a^Os valores se diferenciam em cada variável devido aos dados ignorados não terem sido considerados para a análise.

^b^Variáveis que continuaram significativas após análise multivariada.

Fizeram VDRL ao nascer 332 crianças (100%), hemograma 289 (87,0%), citologia/proteína do LCR 219 (66,7%), VDRL do LCR 219 (66,2%) e RX de ossos longos 260 (80,2%). O peso médio da coorte foi de 3.160 g, variando de 940 g a 5.160 g. Nasceram com baixo peso (< 2.500 g) 37 (11,2%) crianças. Em relação à idade gestacional ao nascimento, 30 (9,1%) foram pré-termo (< 37 semanas), sem associação significativa com o desfecho de seguimento.

Na análise de regressão logística multivariada, ter mais de uma gestação, não ter feito RX de ossos longos e ter resultado do hemograma alterado mantiveram-se implicados no desfecho seguimento não adequado da criança notificada com sífilis congênita.

## DISCUSSÃO

Podem-se observar com este estudo problemas graves em relação ao seguimento ambulatorial de crianças notificadas com SC. Proporção considerável compareceu à unidade de atenção primária, porém a maioria não teve a SC investigada nesse nível de atenção. Tal fato demonstra a pouca visibilidade e reconhecimento da SC como um problema importante de saúde pública, seja por falta de sensibilização dos profissionais ou em razão de os mesmos acharem que o seguimento dessas crianças não é atribuição da atenção primária.

No município de Fortaleza, a atenção primária funciona por meio da Estratégia Saúde da Família (ESF), e a grande maioria das gestantes com sífilis frequenta o pré-natal nessas unidades, para onde, muito provavelmente, levam o bebê para seguimento. Chama atenção que os profissionais não tenham considerado a história pregressa da mãe, seu diagnóstico de sífilis na gestação e o histórico do nascimento da criança, demonstrando perdas de oportunidades na avaliação para a prevenção das formas graves de SC.

Mesmo com o comparecimento das crianças a algum serviço de saúde, foi alta a proporção de não adesão ao seguimento, situação evidenciada também em outros estudos sobre esta temática^13^ . As pesquisas que avaliaram o seguimento de crianças com SC foram realizadas em ambulatórios de referência e não consideraram os motivos da baixa adesão. Acredita-se que a distância da unidade à residência tenha contribuído sobremaneira para a ausência das crianças nesses serviços.

Evidenciou-se que as crianças também não retornaram para continuar o seguimento nas unidades de atenção primária à saúde. É possível que as mães tenham procurado inicialmente essas unidades para realizar a puericultura e os profissionais tenham perdido a oportunidade de encaminhar adequadamente os casos. A baixa adesão à puericultura pode ter ocorrido em função de as crianças não apresentarem problemas de saúde que justifiquem, para a mãe, a necessidade de comparecer frequentemente à unidade. Parece existir uma ideia equivocada de que, por não apresentarem sintomas, as crianças notificadas com SC não precisam de acompanhamento, situação corroborada por um estudo que avaliou a puericultura no Sul e no Nordeste do Brasil e descobriu que o acompanhamento adequado das crianças ocorreu somente em 20% dos casos^17^ .

A baixa adesão ao seguimento poderia ser minimizada se os profissionais de saúde, tanto na ocasião da alta hospitalar como no momento do comparecimento da criança à primeira consulta, tivessem o cuidado de orientar as mães acerca de sua importância, reforçando o comparecimento às consultas subsequentes, especialmente no caso da SC, em que a maioria das crianças nasce assintomática.

Tais achados remetem à necessidade de refletir acerca da organização da rede de atenção à saúde, especialmente no que diz respeito à definição dos locais apropriados para acompanhar as crianças com SC após a alta hospitalar. Faz-se necessário definir o papel da atenção primária nesse processo, bem como sensibilizar e capacitar os profissionais nesse nível de atenção.

À época desta pesquisa, todas as crianças com SC, na ocasião da alta, eram encaminhadas aos ambulatórios de referência das maternidades. Desde a realização deste estudo, essa situação vem se modificando e, atualmente, o município discute a organização da rede de assistência com definição dos locais apropriados para o seguimento dessas crianças e garantia do atendimento, da busca ativa e da referência e contrarreferência.

O município de Fortaleza adota como política pública a vinculação de todas as crianças a uma unidade de atenção primária. Elas devem receber visita domiciliar de profissionais da ESF com o intuito de orientar as mães acerca da importância do comparecimento às consultas e avaliar a adesão às recomendações, entre outros objetivos^18^ . Vale destacar que, mesmo acompanhadas em unidades de referência, as crianças com SC devem ser monitoradas pelos profissionais da atenção primária.

Neste estudo observou-se que, tanto nos ambulatórios das maternidades quanto na atenção primária, a média de consultas ficou abaixo do recomendado pelo MS para a SC, que são nove consultas no primeiro ano de vida^12^ . Em Guarapuava (PR) o seguimento de 40 crianças expostas ou diagnosticadas com SC teve uma média de 6,1 consultas em um ano^19^ .

A idade média das crianças quando apresentaram dois VDRL negativos consecutivos foi de 3,8 meses. Esse resultado era esperado, uma vez que, nas crianças adequadamente tratadas, o VDRL tende a ficar negativo entre os três e seis meses de idade^12^ . Em Porto Alegre (RS), um estudo avaliou o seguimento de 119 crianças acompanhadas com diagnóstico de SC e detectou que, em 81,5%, o VDRL tornou-se não reagente aos três meses de idade^13^ .

É importante identificar os aspectos sociodemográficos maternos que podem impedir o comparecimento das crianças ao serviço. Filhos de mães solteiras retornaram menos para o seguimento, o que pode ser atribuído ao fato de elas serem as provedoras do lar e encontrarem dificuldades para se ausentar do trabalho, ou por outros motivos que as impedem de acompanhar os filhos às consultas, como falta de recursos financeiros.

Foi observada associação significativa entre o número de consultas de pré-natal e a baixa adesão ao seguimento. No caso das mães que não aderem ao pré-natal, torna-se esperado que também não compareçam ao seguimento da criança. Portanto, considerando que as unidades de atenção primária de Fortaleza atuam na lógica da ESF, os profissionais deveriam ter dado atenção redobrada à realização da busca ativa dessas mulheres e seus filhos.

Uma situação preocupante foi que a totalidade das crianças expostas concomitantemente à sífilis e ao HIV não realizaram seguimento, o que deveria acontecer no ambulatório especializado, com monitoramento da atenção primária. As crianças expostas ao HIV tendem a receber maior atenção por parte dos serviços, situação evidenciada em uma pesquisa realizada em São Paulo (SP), que encontrou maior adesão ao acompanhamento dessas crianças do que daquelas expostas e/ou diagnosticadas com SC^14^ .

Mulheres com mais de uma gestação apresentaram maior chance de não realizar acompanhamento dos filhos, situação semelhante à encontrada em Curitiba (PR)^15^ . Ter mais de um filho parece dificultar a ida da mulher aos serviços, seja por outros afazeres domésticos ou pela necessidade de encontrar alguém que possa ficar responsável pelas outras crianças durante a sua ausência.

Alterações no hemograma e a não realização da RX de ossos longos apresentaram associação estatisticamente significativa com o não seguimento das crianças, situação que demonstra falhas graves na efetiva vinculação delas aos serviços. Esses casos podem apresentar efetivamente diagnóstico de SC e, portanto, merecem maior atenção e orientação, garantia de agendamento da consulta e busca ativa em caso de não comparecimento.

A sífilis congênita tardia é decorrente da SC precoce não tratada ou não curada em crianças acima de dois anos de vida^12^ . No presente estudo, as crianças observadas com atraso no DNPM no grupo que não aderiu ao seguimento podem apresentar sequela tardia da sífilis.

Algumas limitações deste estudo estão relacionadas ao fato de terem sido analisados dados secundários, devido à falta de registros e informações incompletas de algumas variáveis. Além disso, não foram excluídas outras condições clínicas que apresentam sinais e sintomas semelhantes à SC, reforçando a importância de mais estudos sobre a evolução dos achados clínicos em crianças notificadas com SC.

## CONCLUSÃO

O seguimento não adequado da sífilis congênita está relacionado não só com a atitude das mães de levar os seus filhos para o atendimento médico, mas também com a dificuldade na realização de exames. A maioria das crianças notificadas com SC comparecem a uma unidade de atenção primária, porém nesse nível da atenção não são seguidas as recomendações do MS para o seguimento adequado. Faz-se necessário melhorar o sistema de referência e contrarreferência entre os diferentes níveis de atenção à saúde, bem como a sensibilização e capacitação dos profissionais de saúde, tanto para o atendimento adequado como para um aconselhamento mais contundente sobre a responsabilidade da mãe com a saúde de seu bebê.
